# Synergistic Herb-Herb Interaction of the Antinociceptive and Anti-Inflammatory Effects of *Syzygium aromaticum* and *Rosmarinus officinalis* Combination

**DOI:** 10.1155/2021/8916618

**Published:** 2021-11-11

**Authors:** Myrna Déciga-Campos, Karla Lizet Beltrán-Villalobos, Hidemi Aguilar-Mariscal, María Eva González-Trujano, Guadalupe Esther Ángeles-López, Rosa Ventura-Martínez

**Affiliations:** ^1^Sección de Estudios de Posgrado e Investigación, Escuela Superior de Medicina, Instituto Politécnico Nacional (IPN), Plan de San Luis y Díaz Mirón S/N, Col. Casco de Santo Tomás, 11340 Ciudad de México, Mexico; ^2^División Académica de Ciencias de La Salud, Universidad Juárez Autónoma de Tabasco, Av. Gregorio Méndez Magaña 2838-A, Col. Tamulte, 86150 Villahermosa Tabasco, Mexico; ^3^Laboratorio de Neurofarmacología de Productos Naturales, Dirección de Investigaciones en Neurociencias, Instituto Nacional de Psiquiatría “Ramón de La Fuente Muñiz”, Calz. México-Xochimilco 101, Col. San Lorenzo Huipulco, 14370 Ciudad de México, Mexico; ^4^Departamento de Farmacología, Facultad de Medicina, Universidad Nacional Autónoma de México (UNAM), Av. Universidad No. 3000, Col. Copilco Universidad, Ciudad Universitaria, 04510 Ciudad de México, Mexico

## Abstract

The use of alternative medicine to treat pain has been increased, and the combination of several medicinal plants for its relief is a common practice in traditional medicine. The present study is aimed at determining whether a combination of *Syzygium aromaticum* (*S. aromaticum*) and *Rosmarinus officinalis* L. (*R. officinalis*) potentiates their antinociceptive and anti-inflammatory effects. These effects were explored using the formalin and carrageenan assays in rats, respectively. Animals received local pretreatment with *S. aromaticum* oil or *R. officinalis* ethanolic extract (0.1–100 *μ*g/paw) alone or combined in a 1 : 1 rate. Concentration-response curves were built to compare pharmacological responses after an individual administration of *S. aromaticum*, *R. officinalis*, or their combination. The pharmacological interaction was investigated by an isobolographic study using the EC_50_ of each component in a fixed 1 : 1 ratio. *S. aromaticum* and *R. officinalis* administered alone showed significant and concentration-dependent antinociceptive and anti-inflammatory effects, but *R. officinalis* was more potent than *S. aromaticum* in both the antinociceptive and anti-inflammatory effects (EC_50_ = 7.96 ± 0.6 *μ*g/paw vs. EC_50_ = 41.6 ± 1.7 *μ*g/paw; EC_50_ = 1.97 ± 0.3 *μ*g/paw vs. EC_50_ = 26.9 ± 2.5 *μ*g/paw, respectively). The isobolographic analysis of the combination of these species in a 1 : 1 ratio showed a synergistic interaction between *S. aromaticum* and *R. officinalis* since *Z*_mix_ (experimental value) was lower than *Z*_add_ (theoretical value) for both the antinociceptive effect (*Z*_mix_ = 0.45 ± 0.1 < *Z*_add_ = 24.8 ± 1.3) and the anti-inflammatory effect (*Z*_mix_ = 5.2 ± 0.6 < *Z*_add_ = 14.4 ± 2.2), suggesting a potentiation for both pharmacological effects. These results prove evidence of the efficacy of mixture herb-herb used in folk medicine for pain therapy. It also emphasizes the requirement of pharmacological studies to explore the efficacy and safety of herb interactions.

## 1. Introduction

Alternative medicine includes medicinal preparations containing minerals, vitamins, nutritional supplements, herbs, and/or homeopathic medicines that interact with other [1]. The use of medicinal plants in combination is widespread in traditional medicine; sometimes two or more extracts, oils, or infusions are combined with the supposition that the therapeutic effect will be increased, while adverse effects will be reduced compared to conventional drugs [2]. It is a common belief that herbal remedies are safe because they are “natural.” However, the use of many of these combinations is empirical because there is no scientific evidence about their benefits, for example, if the mixtures proportions are adequate, if the type of interaction that these combinations produce is good, or if their adverse effects are diminished.

There are a lot of scientific studies about the herb-drug interactions [3], but there are very few scientific studies related to herb-herb interaction despite the fact that this is a common practice in traditional medicine. Although as in the drug-drug or herb-drug interaction studies, the main purpose of combining medicinal plants is to increase their efficacy and to decrease the doses. Furthermore, it is necessary to know if the interaction of natural products could also induce synergism, addition, or antagonism of a therapeutic or even toxic response [2, 4]. Thereby, to find the correct proportion that produces a better therapeutic effect will permit the design of an adequate phytoformulation.

In this study, we selected *Syzygium aromaticum* (*S. aromaticum*) and *Rosmarinus officinalis* L. (*R. officinalis*) as two herbs commonly used in aromatherapy for their analgesic and anti-inflammatory properties [5, 6] to find out if their combination improves these effects. On the one hand, *R. officinalis* (Lamiaceae), popularly named rosemary, is an evergreen perennial shrub that has shown anti-inflammatory, antinociceptive, and antiarthritic effects in rodents using different experimental models [7]. According to various studies, the antinociceptive and anti-inflammatory effects induced by rosemary are mediated by the COX-2 and 5-lipoxygenase inhibition [8, 9], while, on its antinociceptive activity, the 5-HT_1A_ and opioid receptors are involved [10]. On the other hand, *S. aromaticum*, popularly named clove, is a plant 10–20 cm tall found in tropical climates [11]. Clove has been used as an antispasmodic and carminative which improve peristalsis; it also has shown antimicrobial and antiviral activity. Its essential oil is used to relieve toothache and inflammation in the mouth [12]. Its anti-inflammatory effect has been related to the inhibition of COX-2 and 5-lipoxygenase, as well as the suppression of the NF-*κ*B [13, 14]. The eugenol is the main compound of *S. aromaticum*, and it is related to the blockage of the transient receptor potential vanilloid (TRPV) [15] and inhibition of the Ca^2+^ currents high voltage (HVACC) in primary afferent neurons [16].

In a preliminary study, it was demonstrated that *R. officinalis* or *S. aromaticum* showed synergistic antinociceptive interaction in coadministration with ketorolac [17]; however, there are not studies assessing the possible interaction between these two medicinal plants. Therefore, the purpose of the present study was to determine the antinociceptive and anti-inflammatory interactions between *S. aromaticum* and *R*. *officinalis* in local administration in rats using an isobolographic analysis.

## 2. Materials and Methods

### 2.1. Compounds and Preparation of Ethanol Extract of *R. officinalis*

The essential oil of *S. aromaticum*, Tween 80, and 37% formaldehyde solution were purchased from Sigma-Aldrich (St. Louis, MO, USA), while the ethanol extract of *R. officinalis* was prepared by maceration following the techniques previously described in our group [17]. *R. officinalis* L. was collected in the State of Morelos, Mexico (voucher specimen, IMSSM-15005 identified by MSc Abigail Aguilar). Briefly, the aerial parts of *R. officinalis* were cut into small bits (330 g) and kept in a container; an extraction was carried out by successive maceration at room temperature (22°C ± 1) for 48 h. The first extraction with hexane (1200 ml × 3) was performed followed by filtration. The residue was extracted with absolute ethanol (1200 ml × 3) and discarded after filtration. The final filtrate was concentrated under a vacuum to eliminate the solvent. The final product yielded 111 g of a green solid ethanol extract (33.6%) [7].

The ethanol extract of *R. officinalis* and the essential oil of *S. aromaticum* were analyzed and characterized as previously described by spectroscopic techniques [17]. Both were suspended in the vehicle (0.5% Tween 80 in 0.9% isotonic saline solution) and prepared on the day of the experiments to their local administration.

### 2.2. Animals

One hundred thirty-two female Wistar rats (180–200 g) were obtained from the Bioterium of the División Académica de Ciencias de la Salud, Universidad Juárez Autónoma de Tabasco. Animals were housed in a climate- and light-controlled room in a light/dark cycle (light from 7 : 00 AM to 6 : 00 PM), under 27 ± 0.5°C, and humidity-controlled conditions free access to standard rodent diet and drinking water before experimental procedures. The experimental protocol was approved by the local institutional animal care (project 20180861) and by the local Ethic and Research Committees of the Faculty of Medicine of Universidad Nacional Autónoma de México (UNAM) (FM/DI054/2018). All experimental procedures followed the recommendations of the Committee for Research and Ethical Issues of the International Association for the Study of Pain, the Guidelines on Ethical Standards for Investigations of Experimental Pain in Animals [18], and the Official Mexican Standard (Mexican Official Norm for Animal Care and Handling, NOM-062-ZOO-1999). Animals were used only once and sacrificed in a CO_2_ chamber immediately after the formalin or carrageenan tests.

### 2.3. Behavior Evaluation

#### 2.3.1. Formalin Test

In the first series of experiments, the antinociceptive effect of treatments was evaluated in the formalin test [19]. Briefly, rats were placed in an open Plexiglass cylinder for 30 minutes to allow them to acclimate to their surroundings. A mirror was placed behind the cylinder to enable observation from all angles. Nociception was induced by the subcutaneous injection of 50 *μ*L of 1% formalin solution into the dorsal surface of the right hind paw. The flinches behavior induced by formalin was quantified at 1-minute intervals every 5 minutes until 60-minutes. It is well known that formalin induced a biphasic response; an initial short acute phase, known as neurogenic phase (Phase I, 0–10 minutes), followed by a prolonged tonic response, known as inflammatory phase (Phase II, 15–60 min). Total nociception was represented as the area under the curve (AUC) of the two phases.

#### 2.3.2. Carrageenan Test

In the second series of experiments, the anti-inflammatory effect of treatments on carrageenan-induced paw edema model wase studied in other groups of rats [20]. Briefly, paw edema was induced by intraplantar (ipl) injection of 50 *μ*L of 1% carrageenan suspension into the right hind paw of rats. The edema (mL) of the administered paw was measured by volume displacement using a plethysmometer (MOD 7150, UGO Basile, Italy) and compared with the volume displaced of the same paw before the carrageenan administration. The increase in the carrageenan-induced paw edema was considered as an inflammatory response and was registered at 1, 2, 3, 4, 5, and 6 h after carrageenan administration. Percentage of inflammation in each time was calculated considering the maximal inflammation induced by carrageenan at 6 h. Total inflammation was represented as the area under the curve (AUC) of the temporal course.

### 2.4. Experimental Design

In this study, we used logarithmic concentrations of ethanol extract of *R. officinalis* (0.1, 1, 10, 30, and 100 *μ*g/paw) and of essential oil of *S. aromaticum* (0.1, 1, 10, 30, and 100 *μ*g/paw) to build their concentration-response curves (CRC). The concentration selection was based on our previous reports [7, 17]. Both were suspended in the vehicle (0.5% Tween 80 in 0.9% isotonic saline solution) and locally administered according to the test in a volume of 50 *μ*L each dose. In the formalin test, treatments were injected subcutaneously in the dorsal right hind paw fifteen minutes before the formalin and the number of flinches was quantified at 1-minute intervals every 5 minutes until 60 minutes. The AUC of each treatment was obtained and a decrease in the number of flinches induced by the treatments represented an antinociceptive effect. The CRC from *R. officinalis* and *S. aromaticum* of their antinociceptive were built to determine their effective concentration 50 (EC_50_). In the carrageenan test, treatments were intraplantarly (ipl) administered fifteen minutes before the carrageenan injection, into the right hind paw of rats, in the same place to carrageenan injection. The AUC of each treatment was obtained and a decrease in the percentage of inflammation induced by each treatment was considered as anti-inflammatory effect. The CRCs to the anti-inflammatory effect induced by from *R. officinalis* and *S. aromaticum* in individual administration were built to determine their EC_50_. In all cases, 0.5% Tween 80 in 0.9% isotonic saline solution were used as vehicle.

An isobologram was constructed to study the antinociceptive interaction and another to study the anti-inflammatory interaction between *R. officinalis* and *S. aromaticum* using their respective values of EC_50_ for each pharmacological effect. With the values of EC_50_ to antinociceptive or anti-inflammatory effect of each vegetal specie, the experimental combinations were calculated at a 1 : 1 rate (*f* = 0.5) using the mathematical method of Tallarida et al. [4]. Concentrations used in combination of each vegetal specie to build the CRC of combination (1 : 1 rate) and to identify the interaction of each pharmacological effect are shown in Table 1.

### 2.5. Statistical Analysis

Data are expressed as the mean ± standard error of the mean (SEM) for each experimental group (*n* = 6). The temporal courses of the average cumulative time of flinches/min or percentage of inflammation/hour were constructed as a function of time in formalin and carrageenan tests, respectively. The CRCs expressed as % antinociception or anti-inflammation were obtained from the area under the curve (AUC) of temporal courses calculated by the trapezoid method in the respective test. In the formalin test, the antinociceptive effect of each treatment was calculated with the sum of AUC of neurogenic phase (phase I) plus AUC of inflammatory phase (phase II). The % effect (antinociceptive or anti-inflammatory) was calculated with the following formula:

(1) $$\begin{array}{c} \%\text{ effect}=\frac{\left({\text{AUC}}_{\text{VEH}}-{\text{AUC}}_{\text{TRAT}}\right)}{{\text{AUC}}_{\text{VEH}}}\times 100. \end{array}$$

A linear sigmoid model was used to determine the EC_50_ of antinociceptive and anti-inflammatory effects of each vegetal specie. In this study, to compare statistical differences in efficacy and potency to both pharmacological effects, we used Student's *t*-test for independent samples. Also, a data analysis for multiple comparisons of the different treatments (*R. officinalis*, *S. aromaticum* or their combination) were made against the group that only received vehicle. For this, we used a one-way analysis of variance (ANOVA) followed by a Dunnett post hoc test. In all cases, differences with a *P* value <0.05 were considered statistically significant. For statistical analyses, the GraphPad Prism 5 (GraphPad Prism 5.0 Software Inc., La Jolla, CA, USA) was used.

To determine the pharmacological interaction of combination of both species on antinociceptive or anti-inflammatory effects, the *Z* additive (*Z*_add_) and *Z* experimental of mixture or combination (*Z*_mix_) values of each pharmacological effect were calculated according to Tallarida et al. [4]. The *Z*_add_ is the theoretical value that corresponds to a sum of individual treatments, while *Z* mixture (*Z*_mix_) is the experimental value obtained with the combination between *R. officinalis* and *S. aromaticum* considering the EC_50_ of 1 : 1 rate. The *Z*_add_ of each pharmacological effect was compared with respect to *Z*_mix_ using Student's *t*-test: if *Z*_mix_ < *Z*_add_ a synergistic interaction is present, if *Z*_mix_ = *Z*_add_ represents additive interaction, and if *Z*_mix_ > *Z*_add_ represents antagonist interaction [4, 21].

## 3. Results

### 3.1. Antinociceptive Activity of *R. officinalis* and *S. aromaticum* in Individual Administration in the Formalin Test

**Figure 1:**
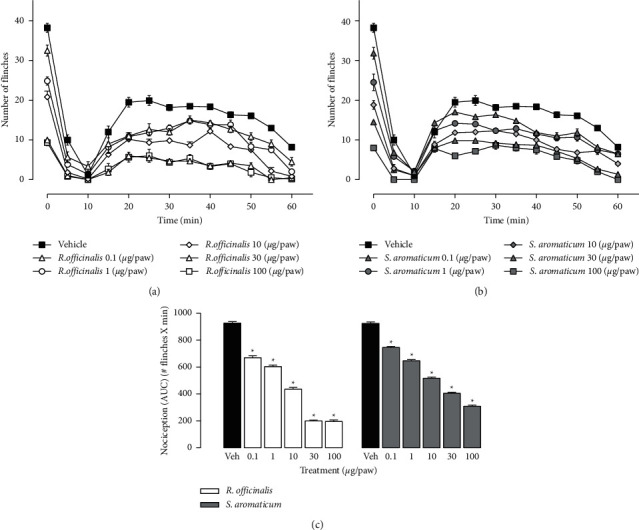
(a, b) Temporal courses of number of flinches induced by the injection of 50 *μ*L 1% formalin into the dorsal surface of the right paw of the rats. The flinches induced by formalin were counted every 5 minutes for 1 minute to complete 60 minutes of evaluation in the presence of *R. officinalis* (a), *S. aromaticum* (b), or vehicle (Veh). All treatments were injected into the dorsal surface of the right paw of the rats 15 minutes before formalin. Each point represents the average of 6 animals tested ± SEM. (c) AUCs obtained from temporal courses of number of flinches in the presence of *R. officinalis*, *S. aromaticum*, or Veh. Each bar represents the mean AUC of 6 animals ± SEM. ^*∗*^Significant difference from vehicle group (*P* < 0.05) as determined by a one-way ANOVA followed by Dunnett's test.

The number of flinches induced with formalin in the vehicle group (Veh) was 38.25 ± 1.1 in the first minute and decreased to 1.16 ± 0.32 at the 10 minutes, when the neurogenic phase (phase I) ended and the inflammatory phase (phase II) began. The maximal number of flinches in the inflammatory phase was 19.9 ± 1.3 at 25 minutes after formalin administration, which remained unchanged until the 50 minutes to later decrease to 8.16 ± 0.7 at 60 minutes (Figure 1). The number of flinches diminished gradually in both phases of formalin test in animals receiving *R. officinalis* ethanol extract (Figure 1(a)) or *S. aromaticum* essential oil (Figure 1(b)). The analyses of the AUCs showed that all doses of *R. officinalis* ethanol extract (0.1, 1.0, 10, 30, and 100 *μ*g/paw) and *S. aromaticum* (0.1, 1.0, 10, 30, and 100 *μ*g/paw) diminished significantly the number of flinches induced by formalin in comparison to vehicle group (Figure 1(c)). This diminution in the number of flinches induced by both vegetal species was interpreted as antinociceptive effect.

### 3.2. Anti-Inflammatory Activity of *R. officinalis* and *S. aromaticum* in Individual Administration in the Carrageenan Test

**Figure 2:**
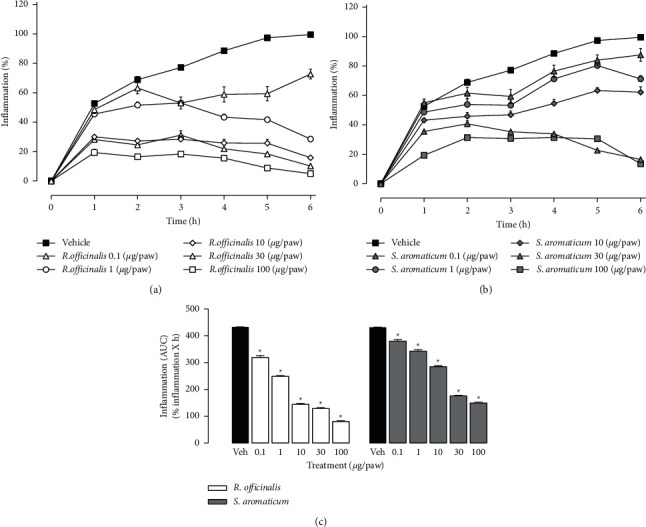
(a, b) Temporal courses of inflammation (%) induced by intraplantar injection of 50 *μ*L 1% carrageenan in the right paw of the rats. Carrageenan-induced edema was measured each hour for 6 hours in the presence of *R. officinalis* (a), *S. aromaticum* (b), or vehicle (control group). All treatments were intraplantar injected in the right paw of the rats 15 minutes before carrageenan. Each point represents the average of 6 animals tested ± SEM. (c) AUCs obtained from temporal courses of % inflammation in the presence of *R. officinalis*, *S. aromaticum*, or Veh. Each bar represents the mean AUC of 6 animals ± SEM. ^*∗*^Significant difference from the vehicle group (*P* < 0.05) as determined by a one-way ANOVA followed by Dunnett's test.

Carrageenan induced a gradual increase of paw edema (mL) in the vehicle group that reached the maximum value 6 hours after its administration (99.5 ± 0.34%) (Figure 2). The paw edema diminished gradually in animals that received *R. officinalis* ethanol extract (Figure 2(a)) or *S. aromaticum* essential oil (Figure 2(b)). The analyses of the AUCs showed that all doses of *R. officinalis* ethanol extract (0.1, 1.0, 10, 30, and 100 *μ*g/paw) and *S. aromaticum* (0.1, 1.0, 10, 30, and 100 *μ*g/paw) diminished significantly the edema induced by carrageenan in comparison to vehicle group (Figure 2(c)). This diminution in the paw edema induced by both vegetal species was interpreted as anti-inflammatory effect.

### 3.3. CRCs of the Antinociceptive and Anti-Inflammatory Effects of *R. officinalis* and *S. aromaticum* and Their Combination

**Figure 3:**
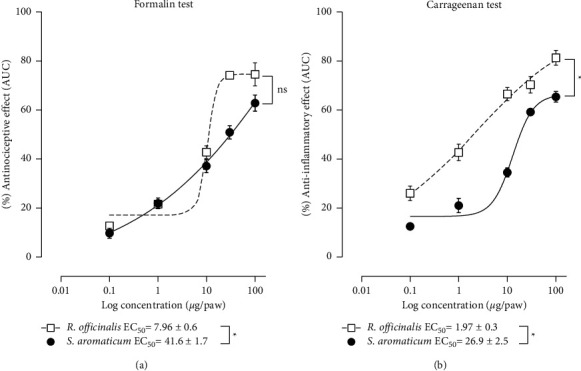
Curve dose-responses of antinociceptive effects of *R. officinalis* and *S. aromaticum* administered individually in different groups of animals in the formalin test (a). Curve dose-responses of anti-inflammatory effects of *R. officinalis* and *S. aromaticum* administered individually in different groups of animals in the carrageenan test (b). Each point represents the mean AUC of the time courses of the number of flinches or edema volume in percentage of effect of 6 animals ± SEM, respectively. ^*∗*^Significant difference (*P* < 0.05) as determined by Student's *t*-test for independent samples.

In the analysis of the CRCs of the antinociceptive effect of both medicinal plants in the formalin test, both showed a similar efficacy since the major effect observed with *R. officinalis* ethanol extract at the highest concentration evaluated (100 *μ*g/paw) was not statistically different with respect to that with *S. aromaticum* essential oil at the same dosage (74.5 ± 4.7 vs. 62.8 ± 3.3%, respectively). Nevertheless, when comparing their EC_50_, *R. officinalis* ethanolic extract showed more antinociceptive potency than *S. aromaticum* essential oil (7.96 ± 0.6 vs. 41.6 ± 1.7 *μ*g/paw, resp., *P* < 0.05) (Figure 3(a)).

Regarding the anti-inflammatory effect, the analysis of the CRCs showed that *R. officinalis* ethanolic extract produced a major effect than that of *S. aromaticum* essential oil at 100 *μ*g/paw, the highest concentration evaluated for each species (81.3 ± 3.1 vs. 65.3 ± 2.2%, resp., *P* < 0.05). Also, when comparing their EC_50_, *R. officinalis* ethanolic extract showed more potency than *S. aromaticum* essential oil (1.97 ± 0.3 vs. 26.9 ± 2.5 *μ*g/paw, resp., *P* < 0.05) (Figure 3(b)).

**Figure 4:**
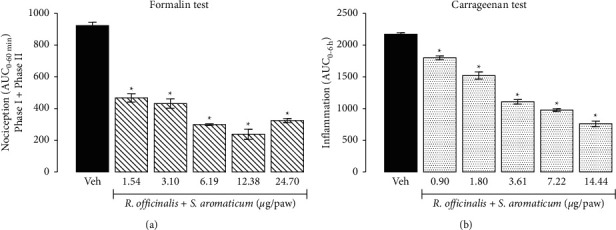
Effect of combination of *R. officinalis* + *S. aromaticum* in fixed-ratio mixture (1 : 1) based on the ED_50_ values of each individual specie on the nociception induced by formalin (a) and on the inflammation induced by carrageenan (b) in different groups of animals. Each bar represents the mean AUC of the time courses of the number of flinches (phase I + phase II) or edema volume of 6 animals ± SEM, respectively. ^*∗*^Significant difference from vehicle group (*P* < 0.05) as determined by a one-way ANOVA followed by Dunnett's test.

After the analysis of antinociceptive and anti-inflammatory interaction of the combination of *R. officinalis* ethanol extract with *S. aromaticum* essential oil, the effect of several combinations in fixed-ratio mixture (1 : 1) showed that all combinations (Table 1) diminished significantly nociception induced by formalin (Figure 4(a)), as well as inflammation induced by carrageenan (Figure 4(b)).

### 3.4. Analysis of Isobolograms of Antinociceptive and Anti-Inflammatory Effect of *R. officinalis* with *S. aromaticum*

**Figure 5:**
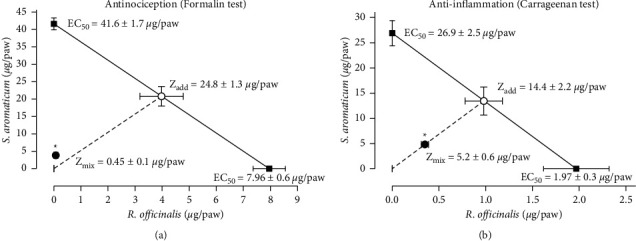
Isobolographic analysis of the antinociceptive (a) and anti-inflammatory (b) effect of S *aromaticum* and *R. officinalis* in combination (1 : 1 ratio). Data represented as mean ± SEM of six rats evaluated in the formalin or carrageenan test in rats. The continuous line represents additivity for all the additive theoretical combinations. The point (○) in the line represents the theoretical additive point, which was calculated from the individual drug EC_50_ values, in this case, was named (*Z*_add_). The point (●) is the experimental EC_50_ (*Z*_mix_) indicating significant synergism. ^*∗*^Statistical difference using Student's *t*-test *Z*_add_ versus *Z*_mix_.

In the analysis of the antinociceptive interaction of *R. officinalis* ethanol extract with *S. aromaticum* essential oil, the isobologram showed that the *Z*_add_ (obtained theoretically) was significantly higher than *Z*_mix_ (obtained from the experimental CRC of 1 : 1 ratio combination) (24.8 ± 1.3 vs. 0.45 ± 0.1 *μ*g/paw, *P* < 0.05) (Figure 5(a)). In the same way, in the analysis of the anti-inflammatory interaction of these medicinal plants, the isobologram showed that the *Z*_add_ was also significantly higher than *Z*_mix_ (14.4 ± 2.2 vs. 5.2 ± 0.6 *μ*g/paw; *P* < 0.05) (Figure 5(b)). As in both pharmacological effects *Z*_mix_ was significantly lower than *Z*_add_, a synergistic interaction was present.

## 4. Discussion

The results of this study demonstrate that the *Rosmarinus officinalis* ethanolic extract in combination with *Syzygium aromaticum* essential oil, in the 1 : 1 fraction, induced synergistic interaction in both in its antinociceptive and anti-inflammatory effects in the formalin and carrageenan tests, respectively.

In this study, the *R. officinalis* ethanol extract showed significant antinociceptive and anti-inflammatory effects in rats. These results agree with previous reports in which *R. officinalis* showed antinociception in the writhing and formalin tests in mice, reinforcing their antinociceptive and anti-inflammatory activity by inhibiting both central and peripheral levels of nociception [6, 7, 17]. In the PIFIR test, an inflammatory pain model like the clinical condition of gouty, *R. officinalis* produced an increase in the percentage of functionality index in arthritic rats reinforcing its antinociceptive activity [7]. In the hot plate test, *R. officinalis* produced a significant antinociceptive effect in mice, corroborating that its analgesic properties were mediated at the central level [22]. Also, its anti-inflammatory effect has been evaluated in the carrageenan-induced edema and systemic-inflammation tests using oral or intravenous administration [23, 24]. In this study, we also demonstrated its anti-inflammatory activity but by local administration in the model of plantar edema induced by carrageenan. According to previous studies of our laboratory about the phytochemical of the ethanol extract of *R. officinalis*, this specie showed a composition rich in terpenes as abietane, carnosol, totarol, and sugiol [17]. Other studies had showed that it also contains flavonoids as quercetin, hesperidin, diosmetin, diosmin, luteolin, and apigenin; tannins and saponins; phenolic acids as caffeic and rosmarinic acids; as well as terpenoids as carnosol and rosmanol [10, 25]. Several metabolites isolated from this specie could be responsible of its antinociceptive and anti-inflammatory properties, as hesperidin by the participation on the TRPV1 receptors [26] or carnosol which attenuated the formation of reactive oxygen species, inhibited the 5-lipoxygenase and leukocyte secretion, blocked Ca^2+^ channels in intact polymorphonuclear cells, and inhibited the COX-2 [9].

On the other hand, the antinociceptive effect of *S. aromaticum* essential oil showed in this study using the formalin test agrees with that reported in a preliminary study [17] and with the results reported in the writhing and hot plate tests in mice [5]. In a similar manner, the present data confirmed the anti-inflammatory activity of *S. aromaticum,* as previously reported in the carrageenan-induced edema [5]. In respect to the phytochemical composition of essential oil *S. aromaticum*, previous results of our laboratory showed that it contains eugenol as the mayor component (79.2% of the mixture), *β*-caryophyllene and methyleugenol in medium proportions (almost 12%) as well as humulene and caryophyllene oxide in minor proportion [17]. In other studies, ethanol extract of *S. aromaticum* has showed the presence of flavonoids (kaempferol and quercetin), caffeic, ferulic, ellagic, and salicylic acids [27], as well as eugenol in higher concentrations (70–90%) [27, 28]. However, the essential oil of this plant is the one most frequently used in traditional medicine for the relief of dental pain [27]. It was demonstrated that eugenol, the main component in the essential oil from this specie, inhibited voltage-dependent sodium channel currents independently of TRPV1 receptors, in primary dental afferent neurons using the patch-clamp technique [29]. Administration of eugenol promotes GABA_A_ currents inhibiting trigeminal ganglion neurons and GABA *α*1*β*2*γ*2 subunit expressed in these neurons [30]. Also, methyleugenol, a derived from eugenol, produced an antinociceptive effect in the formalin model due to the inhibition of hyperalgesia through NMDA receptors mediated by the GABA_A_ receptor [31].

After confirming the significant antinociceptive doses of individual administration of *S. aromaticum* and *R. officinalis*, this study explored the results of combined doses of these two medicinal plants. When two or more drugs are coadministered, the selection is based on the fact that different mechanisms could promote a synergistic interaction. However, there is information about two drugs sharing the exact mechanism of action that produced synergism. For example, the combination of metamizole plus paracetamol enhances an antinociceptive response. It is known that both inhibit the same enzyme (cyclooxygenase 1 and 2), but they also possess other mechanisms [32]. In this work, the combination of these medicinal plants induced synergistic interaction in its antinociceptive and anti-inflammatory effects. It is important to mention that a complex mixture of bioactive constituents exerting multiple mechanisms of action could be involved in the effect of a combination of plant extracts. It has been reported that *R. officinalis* significantly reduced COX-2 mRNA expression in the LPS-activated cells [33], in part due to its components rosmarinic acid and rosmanol [8, 34]. In the case of *S. aromaticum*, it also downregulated the expression of COX-2 isoform [35], and its antinociceptive effect has been associated with the participation of *α*_2_-adrenergic and opioid receptors, in which eugenol, its major component could be partially responsible [29, 36]. Concerning inflammation, carrageenan injection produces an inflammatory reaction that mainly depends on the imbalance between the activation of proinflammatory cytokine cascade and the induction of anti-inflammatory cytokines [37]. It has been reported that neutrophils and macrophages may develop inflammatory responses, hyperalgesia, allodynia, edema, and fever, with a consequent increase in several inflammatory mediators released by these cells in carrageenan-induced inflammation [37]. *R. officinalis* extract promotes phosphorylation of MAPKs, thereby blocking NF-*k*B activation, leading to decreased expression of iNOS and COX-2, thus preventing inflammation [33], while the anti-inflammatory effect of extract of *S. aromaticum* could be related to the inhibition myeloperoxidase (MOP) activity [38].

The use of medicinal plants in combination to manage pain could be a good option if the effect of their combination is scientifically proven. For example, there is a study where a famous herbal remedy in China known as Shaoyao Gancao decoction, composed of the roots of paeony (*Paeonia lactiflora*) and licorice (*Glycyrrhiza uralensis*), showed an antinociceptive synergistic interaction in a neuropathic pain model [39]. Another herbal remedy used for migraine is Tou Feng Yu Pill, which is composed of *Radix angelicae dahuricae*, *Rhizoma chuanxiong,* and *Folium camelliae sinensis*. The Tou Feng Yu Pill showed a significant reduction in the number of stretches induced by acetic acid and a decrease in the number of nociceptive behaviors in the formalin test and experimentally reduced migraine [40]. In another study, it was proven that the Tianshu formula, which is the combination of two herbs, *Rhizoma chuanxiong* and *Gastrodia elata*, presents a synergistic interaction that decreased migraine [41].

## 5. Conclusions

In conclusion, a synergistic interaction between *R. officinalis* and S*. aromaticum*, both in the antinociceptive and in the anti-inflammatory effect, was observed in this study, suggesting that this combination of medicinal plants could relieve inflammatory pain associated with several diseases in human beings and reinforcing the importance of studying the pharmacological interactions herb-herb to provide scientific evidence of its usefulness in folk medicine.

## Figures and Tables

**Table 1:** Sum of doses used in the concentration-response curves in the formalin and carrageenan-induced edema tests.

Nociception concentration (*μ*g/paw)			Inflammation concentration (*μ*g/paw)		
*S. aromaticum*	*R. officinalis*	Sum	*S. aromaticum*	*R. officinalis*	Sum
1.30	0.24	1.54	0.84	0.06	0.90
2.60	0.49	3.10	1.68	0.12	1.80
5.20	0.99	6.19	3.36	0.25	3.61
10.40	1.98	12.38	6.73	0.49	7.22
20.80	3.96	24.7	13.45	0.99	14.44

## Data Availability

The data used to support the findings of this study are available from the corresponding author upon request.
